# The theory of integrated empowerment in the transition to adulthood: concepts and measures

**DOI:** 10.3389/fsoc.2023.893898

**Published:** 2023-04-17

**Authors:** Najla Mouchrek, Mark Benson

**Affiliations:** ^1^Department of Art and Design, College of Arts, Media, and Design, Northeastern University, Boston, MA, United States; ^2^Interdisciplinary PhD, Virginia Tech, Blacksburg, VA, United States; ^3^Department of Human Development and Family Science, Virginia Tech, Blacksburg, VA, United States

**Keywords:** theory, empowerment, emerging adulthood, college student development, early adult

## Abstract

**Introduction:**

Developmental challenges in the transition to adulthood require a process of empowerment that enables young people to guide themselves and build capacities for adult commitments and roles. To examine this systemic process, we conducted an interdisciplinary investigation of constructs from prior literatures that relate to empowerment. Two foundational dimensions of empowerment emerged in relation to individual functioning and relational environments.

**Theoretical model:**

The two dimensions are self-direction and meaningful roles in society. A creative process of theory construction informed by related literatures identified four component catalysts that drive these dimensions of empowerment among early adults: personal agency, sense of purpose, mentoring experience, and engagement in community. As developed in this article, the Integrated Empowerment Theory explains the relationships among these catalysts within the ongoing, multilayered process of empowerment in the transition to adulthood. A graphic representation in the article specifies the relationships among these theoretical concepts.

**Method and results:**

To advance future research based on these theoretical concepts, we constructed multi-item measures of the four catalysts drawn from indicators in the empirical literature. The resulting scales were presented to participants in an empirical test of their technical adequacies. Participants were 255 early adult college students from eight colleges at a public land-grant research university in the United States. The 18-item scale includes four subscales: agency, purpose, mentoring, and community. The study findings evidenced robust internal consistency estimates across the scales (0.79–0.96).

**Discussion:**

The Integrated Empowerment Theory and the corresponding scales provide tools for research to understand and promote positive developmental outcomes for youth as they navigate experimentation, life choices, and identity construction. The scales also imply a logical sequence for application and intervention. The sequence corresponds to four key catalysts: Community, Agency, Mentors, and Purpose, or CAMP. Although the conceptualization and the scales draw from a college population, the constructs have potential applicability, and await future research with additional age groups. For early adults, empowerment has particularly important implications for societal contributions. Creating contexts where youth can play meaningful roles in their emerging social world holds positive potential for society.

## 1. Introduction

Social, cultural, and economical changes in Western societies in the last few decades have generated new challenges and opportunities on the road toward adult life. On one side, youth are granted an extended period of transition with more opportunities for experimentation before committing to particular values and roles. On the other side, life paths are increasingly individualized, less structured, and more self-authored, requiring agency, personal meaning-making, and self-reliance. To understand and guide research on these important issues, the field needs theoretical models grounded in relevant, related research literatures.

The purpose of this paper is just that, to focus on the transition to adulthood and advance a theoretical model of empowerment. According to Miller (2016), theoretical models make two important contributions. First, they serve an interpreter role, advancing understanding and addressing the human need for meaning-making. Second, many theoretical models also have the capacity to fill a stimulator role, guiding future research or leading to new technologies. The current paper seeks to make both types of contributions. First, toward advancing interpretation, the paper draws concepts from multiple theoretical and research literatures that relate to youth empowerment. These concepts provide the elements for a set of constructs representing essential dimensions of youth empowerment.

The paper also seeks to serve the second role, that of stimulator, for both research and for application. To aid future research, we present four measures of the constructs advanced in the theory model. In support of use with future research, we show the item-content and findings from our pilot research with four scales, which exhibited robust internal consistency estimates (0.89–0.94). These scales also support testing empowerment applications, as the brevity of these measures with 4 to 6 items each facilitates use in repeated-measures research examining the interventions and applications that are influenced or inspired by the theoretical model.

Although the theoretical model is potentially applicable to a wide range of adults, the focus here is on college students based on the potential of universities to constitute, by design, empowering settings for youth growth and development in this life stage.

## 2. Development in the transition to adulthood

During the college years, students are navigating the complex transition from adolescence to adulthood. Marked by developmental challenges, this period may also constitute a time of opportunity for establishing positive self-identities and shaping individual futures, both personal and professional. In this phase, the young person can develop competencies, attitudes, values and maturity necessary to a meaningful and successful transition into adulthood.

The process of identity formation and preparation for commitment to specific values and adult roles is an essential developmental task in this life stage. Fundamental changes during adolescence across multiple domains (biological, cognitive, psychological, and social) instigate a process of social and interpersonal redefinition characterized by increasing levels of autonomy (Steinberg, 2007). Identity development and search for understanding one's unique role in the world were conceptualized by Erikson's Psychosocial Theory (Erikson, 1950, 1968) as the core existential motivations in late adolescence. This theory explains why youth is essentially a phase of experimentation, characterized by a desire to explore different identities and ways of living, in search of values and models that are meaningful for them.

Advancing Erikson's theory, Marcia (1966, 1993) proposed four identity development statuses, according to the combination of two basic dimensions of exploration and commitment: moratorium (young person is exploring life options, but still not ready to commit to a defined set of values and identity), foreclosure (early commitment without sufficient exploration, usually led by external factors), achievement (identity resolution, with a balance of exploration and commitment), and diffusion (extended exploration without commitment, leading to a diffused identity and lack of sense). Since identity development is a dynamic process, people may move among these statuses. An initial status of foreclosure, for example, may lead to achievement if the person undertakes exploration through a period of moratorium. According to Meeus (2011), the identity status continuum will likely follow a typical pattern in the order: diffusion → moratorium → foreclosure → achievement.

More recently, Luyckx et al. (2006) added two dimensions to Marcia's framework: exploration in depth and identification with commitment (both involving continuous revision of existing commitments). Later, Luyckx et al. (2008) further advanced the model by adding ruminative exploration as a new dimension representing a maladaptive identity process (repeated cycles of exploration hindering commitment).

When analyzing identity formation in the transition to adulthood, Roberts and Côté (2014) proposed a framework which differentiates: (a) self-identity tasks, including processes of integration and differentiation; (b) social-identity tasks, comprising work roles and worldview. The authors also state that those processes happen simultaneously in three levels of analysis: (a) ego identity, linked to subjective experience; (b) personal identity, linked to behavioral repertoire; and (c) social identity, related to social roles and statuses.

Important transitions in cognitive aspects also take place during this stage. The young person's competencies for decision-making, critical thinking, planning, risk/reward assessment, and creative problem-solving advance due to neurological maturation—as presented in Steinberg (2005) review of research in cognitive and affective development in adolescence; and summarized in Beck (2012) review article. Continued cognitive and neural development and the strengthening of brain pathways lead to enhanced capacities for processing emotional and social information. The development of more advanced, practical, flexible, and dialectical cognitive processes is observed (Steinberg, 2005). The young person's sense of self and capacity for self-reflection pass through significant changes. In this period, early adults likely consolidate their worldview, while also recognizing other valid perspectives (Arnett, 2014).

The process of construction of self occurs through experimentation: in adolescence and early adulthood, there is a richness of experience, since it is a period when the brain presents great plasticity and is extremely sensitive to experiences (Erikson, 1968; Steinberg, 2014). Youths are thinking about themselves and the world around them in new and interesting ways; this theoretical thinking is tested through real-world, trial-and-error experimentation. New life experiences are gradually integrated by new advanced thinking skills in personally meaningful ways, resulting in a complex and rich process of identity development (Nakkula and Toshalis, 2006; Steinberg, 2014).

Identity development in adolescence and early adulthood is also a social and cultural construction, consisting of a relationship-based process, in which the interpretation young people make of themselves, and their worlds is essential—as shown by Nakkula and Toshalis's (2006) review on adolescent development research and theory. Experiences of various “possible selves” and an increasing sense of self-understanding and understanding others (as proposed by Harter, 2012; Markus and Kitayama, 2012) may lead to the development of a renewed self-concept and to a broad process of changing roles and modes of interaction with others.

The challenges in the transition to adulthood involve shifts in relationships with parents, exploration of new roles, processes of identity formation, planning about the future, and undertaking steps to achieve personal goals (Eccles and Gootman, 2002). Completing education and entering the job market are two major milestones common to this phase.

### 2.1. Emerging adulthood

In the past decades, the concept of Emerging Adulthood in industrialized societies as an identified developmental era with a focus on ages 18–25 was established (Arnett, 2007, 2014). In contemporary Western societies, demographic changes and transformations in social structures contribute to the emergence of this distinct period of the life course. Young people have an extended period (“moratorium”) for exploration of possible life directions before taking adult commitment.

Emerging adulthood is identified as a phase characterized by identity exploration, cognitive flexibility, feeling in-between, opportunities for transformation, and taking responsibility for one's life (Arnett, 2014; Self-Authorship Theory, Baxter-Magolda, 2002). This is considered a fundamental period in youth development, because it is rich with possibilities in several aspects of life, when many possible futures and personal exploration remain possible.

Evidence from prior studies demonstrate that emerging adulthood may denote an ambivalent developmental stage, being either a source of wellbeing or anxiety and depression for youth (Schwartz, 2016). A review of psychological and sociological theory and research in identity formation in youth (Côté and Levine, 2002) reveal that if, on one side, youths have more freedom to define their career and life paths, on the other side their trajectories are more individualized, less supported by collective processes, and require more responsibility and authorship. Empirical findings from Schwartz et al. (2005) show that, due to increased identity choices and lack of structure, the identity development process in emerging adulthood is often characterized as a personal project requiring agency in order to negotiate the transition to adulthood.

During this phase, people are gradually taking on adult roles in several life domains. However, findings from research (Arnett, 2001; Sharon, 2016) demonstrate that the markers of adulthood are changing and becoming increasingly individualized, and that emerging adults are postponing traditional role transitions like graduation, full-time employment, marriage, and parenthood.

The theoretical framework of emerging adulthood can be particularly valuable to understand the developmental routes and needs of college students. Distinctive features of the North American college experience, in particular, provide a distinctive environment for development in this stage (Arnett, 2016). Studies on developmental trajectories within the college context have the potential to successfully support strategies to promote student development and academic success.

### 2.2. College student development

For many youths, universities play a significant role in the transition to adulthood. Higher education provides a unique opportunity to improve both cognitive and psychosocial development. As mentioned, early adulthood might be considered a key turning point in the life span (Schwartz, 2016). College experiences provide avenues for students to constitute identity and offer opportunities for role experimentation, personal choice, meaningful achievement, and time for reflection.^1^

We highlight the centrality of a holistic inclusive approach to learning and development, concerning physical, mental, emotional, and spiritual factors. In a developmental conceptualization of learning, Baxter-Magolda (1996) states that cognitive learning and personal development are intertwined processes. An early theoretical formulation (Bloom, 1964) sustains that self-development involves the cognitive, affective, and psychomotor domains of learning. Prior theorizing (Chickering and Reisser, 1993) proposes that student development is composed of seven vectors: “achieving competency; managing emotions; moving through autonomy toward interdependence; developing mature interpersonal relationships; establishing identity; developing purpose; developing integrity^2^”. Strategies to promote college students' whole development must consider aspects such as: the need for healthy identity exploration; reflection and experimentation with values and principles; and search for meaning and purpose in professional and personal choices.

Self-authorship was proposed by Baxter-Magolda (2008) as “the internal capacity to define one's beliefs, identity, and social relations” (p. 269). The concept emerged as a core developmental capacity to support youths to face challenges related to adult life. Recent research focusing on college students (Baxter-Magolda and Taylor, 2015) demonstrates that the journey to self-authorship involves “developing internal criteria for crafting one's identities, relationships, and beliefs, yielding the ability to navigate external demands” (p. 299). Creating conditions to promote self-authorship in the knowledge construction process in this stage of life is fundamental.

A useful conceptualization for student development is Kegan's Theory of the Evolution of Consciousness (1994). The author defines the growing process as an unfolding of organizing experiences that are progressively incorporated into more complex systems of mind (as summarized in the theory review by Evans et al., 2009). Kegan's (1994) developmental stages/orders of consciousness are: impulsive mind; instrumental mind; socialized mind; self-authoring mind; and self-transforming mind. Strategies to promote development in college should consider ways to foster evolution through those stages. Undergraduate teaching and advising, for example, may encourage reflection, discussion, and intentional goal setting so that students have opportunities to gradually develop their consciousness.

Another important reference is the work of Pascarella and Terenzini, (1991, 2005; Mayhew et al., 2016), which synthesizes research that spanned decades about the impact of the undergraduate experience in student populations — including cognitive, moral, and psychosocial development, values and attitudes, educational attainment, and personal and professional post-graduation outcomes. Pascarella and Terenzini (1991, 2005) found that college had a positive influence on diverse subject matter and cognitive outcomes, promoting academic and social self-concepts, sense of control, and leadership skills. They found increases in critical thinking, leadership, self-concept, independence from authority, principled moral reasoning, and spirituality. In their third volume (Mayhew et al., 2016), most recent findings partially confirmed those gains and provided a more nuanced and comprehensive analysis with additional insight on how college may promote or hinder student outcomes.

As potential contexts for students' growth and empowerment, universities should design the college experience having in mind the perspective of youth developmental needs. It matters to capture students' lived experiences during these years and understand what makes an empowering experience in formal and informal learning settings in college.

## 3. Empowerment in the transition to adulthood

Youth is a time of maximal opportunity for developing positive self-identities and healthy insertion in society. In a phase in which individuals will likely decide on and commit to particular courses of actions in life, room for experimentation and choice helps to navigate the course toward adult commitments and roles. Current literature regarding both theory and research on development in the transition to adulthood (Côté and Levine, 2002; Bynner, 2005; Arnett, 2014; Baxter-Magolda and Taylor, 2015; Schwartz, 2016) shows the heightened importance of fostering self-direction and supporting youth to develop meaningful roles in contemporary society, due to added developmental choices. The transition to adulthood calls for a process of empowerment that supports youth in guiding themselves, envisioning and implementing future development plans.

Experiences of empowerment that surround the early adult have important contributions to positive development. Findings from research in health programs for youth (Chinman and Linney, 1998) showed that empowering experiences led to outcomes such as healthy identity experimentation, gains in confidence, critical awareness, self-efficacy and self-esteem. Identity development and career exploration are key processes requiring empowerment in this period.

### 3.1. Defining empowerment

The concept of empowerment emerged in the field of community psychology, and it was first defined intentional, active, ongoing “process by which people gain control over their lives, democratic participation in the life of their community, and a critical understanding of their environment” (Rappaport, 1987; Cornell Empowerment Group, 1989). In their review of empowerment theory, research, and application, Perkins and Zimmerman (1995) affirm that empowerment is a highly popular concept nowadays, but it is often inadequately conceptualized and loosely defined. Empowerment is the object of multiple conceptualizations: theoretical models and empirical studies have been developed in various fields.

Since the inception, empowerment is recognized as a context- and population- specific construct: both empowerment processes and outcomes vary since it is not possible to define a sole standard to comprehend all richness and variety of its meaning for different people in different contexts and developmental stages (Rappaport, 1984; Zimmerman, 1995). Perkins and Zimmerman (1995) defend that it is fundamental to clearly conceptualize and communicate what empowerment means in each context or in a particular theoretical proposition.

Traditionally, empowerment theory distinguishes three levels of analysis: individual, organizational, and community empowerment. Although positioned as a study of empowerment at the individual level, the present work is also integrative since it recognizes the interdependence of levels and addresses multiple interplaying processes.

We identified the need to build a comprehensive model of developmental empowerment in the transition to adulthood, addressing specific contexts and challenges of early adulthood and integrating inputs from interdisciplinary theories.

### 3.2. Critical analysis of prior conceptualization

This section presents a critical analysis of prior conceptualizations of empowerment relevant to this process of theory construction. A table summary of these theories appears in Appendix A.

When analyzed at the level of the individual, the focus of empowerment is on capacity-building. Psychological Empowerment was conceptualized by Zimmerman (1995, 2000) as composed of three interrelated components: (a) intrapersonal/emotional, believing in one's ability to exert control, perceived competence, efficacy, mastery; (b) interactional/cognitive, the critical awareness and understanding the sociopolitical environment; and (c) behavioral, the individual actions that address needs and directly affect outcomes.

Although Zimmerman's theory addresses relevant components of empowerment at the individual level, it lacks a clear explanation of how the empowerment process actually happens. For more insights to this process, Christens (2012) proposed a theoretical addition to the psychological empowerment model—a relational component. The foundation of this relational component involves principles of mutual support, collaboration, and collective exercise of transformative power to achieve change. Specific elements such as collaborative competences, facilitating empowerment of others, and engagement in participatory behaviors describe the actions of the relational component.

This relational component is a promising addition to the earlier version of psychological empowerment (Zimmerman, 1995, 2000), which was absent from the earlier conception. The more clearly delineated process emphasizes the key role of relationships in empowerment with particular attention to collaboration and mutual growth. The influence of participation in groups is now more evident, with emphasis on the role of mentors and community. However, the model remains limited in providing a deeper understanding of the empowerment process for each individual, considering both personal development and real-life experiences.

The model of Psychological Empowerment (Zimmerman, 1995; Christens, 2012) does not specifically identify the process in transitions across development. By contrast, Chinman and Linney (1998) lay out fundamental concepts of adolescent development and the developmental gains from an empowering process implemented by adults in youth programs. In their Adolescent Empowerment Cycle, a holistic definition for youth empowerment emphasizes opportunities for youth to participate in meaningful roles in community as central factors for experimentation and identity construction. Other key aspects are dynamic partnerships including recognition from adults and approval from peers, and time for self-reflection and personal understanding of experiences.

The Adolescent Empowerment Cycle (Chinman and Linney, 1998) is a foundational work in youth empowerment, bringing essential constructs that would be part of almost all conceptualizations later on (including our own theoretical approach). Grounding their work in the theory of Social Bonding Development (Hawkins et al., 1992), the authors explain how youth participation in activities leads to developmental outcomes. They suggest that the major developmental risk to be avoided is rolelessness—hence the focus in developing meaningful roles for youth. Chinman and Linney (1998) theory contributes to understanding meaningful roles and developmental risks in empowerment. Still, it does not fully explain the dynamic nature of the empowerment process between individual and external environment.

The theoretical framework of Cargo et al. (2003), however, introduces a dynamic model of active community participation. In their model, empowerment is defined as a transactional partnership between adults and youth, in which adults develop social contexts for youth to gradually take responsibilities, while youth will gradually engage, actualize potential, and cultivate constructive change. The authors offer a significant contribution to the understanding of processes of accompaniment and gradual transfer of responsibility between adults and adolescents. The work illuminates the nature of mentoring relationships and the developmental outcomes from intentional processes to promote empowerment among youth. However, the interventions as described by the authors are limited to community engagement programs for adolescents that are planned and implemented by adults. Extended contexts of life and activity, coupled with the variety of mentors and growing autonomy in making meaning and navigating life in early adulthood call for an advanced model of developmental empowerment that captures the interplay of all those elements with the internal, psychological processes.

The framework proposed by Cargo et al. (2003) conceptualized the principles of adult-youth partnerships for empowerment with a developmental approach. Jennings et al. (2006) also adopt a developmental approach, but they center adult-youth interactions within a broader set of community and sociopolitical processes. In proposing their Critical Social Theory of Youth Empowerment, Jennings and colleagues build upon previous work (i.e., Freire, 1970; Chinman and Linney, 1998; Cargo et al., 2003; Wallerstein et al., 2005). And using empirical findings from participatory research, they conceptualize empowerment as a multi-level construct, described as a series of experiences in which “youth, adults, organizations, and communities engage in collective action for social change” (Jennings et al., 2006, p. 52). Their theory identifies six key dimensions: (1) welcoming and safe environment; (2) meaningful participation and engagement; (3) equitable power-sharing between youth and adults, (4) engagement in critical reflection on interpersonal and sociopolitical processes; (5) participation in sociopolitical processes to affect change; (6) integrated individual- and community-level empowerment (p. 32).

The theoretical model proposed by Jennings et al. (2006) encompasses and organizes essential elements of empowerment in previous theories in a critical framework oriented to the potential of young people to participate in and influence social changes. The description of the elements is quite accurate, and the model represents an advance toward a more comprehensive model. However, the framework is not explicit about the dynamic interaction among the elements and restricts the application of empowerment to social change as the outcome.

Cattaneo and Chapman's (2010) model of empowerment contrasts with Critical Social Theory (Jennings et al., 2006) in advancing a dynamic view of empowerment in diverse contexts. Cattaneo and Chapman incorporate both individual and social aspects in conceptualizing empowerment as an iterative process. The key components in their model are: “personally meaningful and power-oriented goals, self-efficacy, knowledge, competence, action, and impact” (p. 646). The authors describe how individuals move through the process focusing on specific goals while reflection derives from experience. Although not positioned as a model of youth empowerment exclusively, Cattaneo and Chapman's model supports understanding dynamic processes of empowerment, as they highlight the role of intrinsic motivation, reflection, and interaction with others. This model contributed to the description of the empowerment process, revealing its iterative and gradually-developing nature. However, the focus on self-efficacy and specific goals does not align with the purposes of the present study, which aims to describe how the empowerment process essentially unfolds in different spheres of development in early adulthood.

The importance of experimentation with different roles in adolescence is explored by Chinman and Linney (1998) theoretical model, but it is not present in other frameworks for youth empowerment. Identity development is briefly mentioned as an empowerment outcome by Jennings et al. (2006). Processes of construction of self are not present in Zimmerman's Psychological Empowerment model (1995) either. either. Earlier models of youth empowerment state the centrality of critical reflection on experiences and critical awareness: while for Jennings et al. (2006) these processes are linked to social change, for Chinman and Linney (1998), they refer to personal development and change (therefore more in tune with our work).

In our process of theory construction, it was essential to consider the interaction between personal and social aspects of development, aiming to advance both youths' personal goals and directedness (nurturing the need for autonomy), and a sense of community and strong interpersonal relationships (nurturing the need for affiliation and acceptance).

## 4. Theory construction: empowerment model

Within the scope of the interdisciplinary research project, we engaged in a process of theory construction, which resulted in a theoretical model of *integrated empowerment in the transition to adulthood*, understood as a process of capacity building for the transition, leading to positive developmental outcomes linked to specific contexts and challenges in early adulthood.

The model focuses on early adults between 18 and 25 years-old, primarily from Western societies (including both developed and developing countries). Our approach is in tune with the original tenets in empowerment theory, as it:

(a) proposes a *holistic model*, addressing people as complete human beings (as in Rappaport's early conceptualization, 1987)
(b) emphasizes the *ecological nature of empowerment*, comprising both determination over one's individual life with psychological sense of control and actual influence in community (as in Rappaport's theoretical formulation, 1981, 1987)—recognizing the mutual influence of these levels
(c) affirms the centrality of *developmental processes that happen in the context of living life*, in settings that offer opportunities to develop and practice skills (as in Zimmerman's conceptualization of psychological empowerment, 1995)
(d) considers empowerment essentially as a *participatory process*, emphasizing the role of relationships, participation in mediating structures in society, and creation of solutions through collaboration [as in the early theories of Rappaport, 1981 and Zimmerman, 1995; and as also affirmed by Maton et al.'s elaboration on empowering settings (Maton et al., 2011)].

The model considers the importance of capturing youth's perspective and lived experiences. Aligned with the positive cultural shift in youth studies,^3^ the present work seeks to recognize young people's uniqueness and potential, providing opportunities for integral development and thriving through participation in creative processes.

The model advances and extends previous theoretical propositions (Chinman and Linney, 1998; Cargo et al., 2003; Jennings et al., 2006; Cattaneo and Chapman, 2010), drawing from interdisciplinary theories, focusing on challenges and opportunities of early adulthood, and emphasizing the nature of empowerment as an interactive, gradual, and multifaceted interplay among developmental experiences at the internal and the external contexts.

Early conceptualizations highlight the importance of empowerment for individuals, organizations, and communities (Rappaport, 1987). Empowerment was defined as affirmation and opportunity to learn and experience growth and development (Rappaport, 1981); and mastery over one's own fate (Rappaport, 1987). However, the emphasis at that time was on interventions to increase the possibilities for people to control their lives *within the community*. In contrast to that, the present approach emphasizes opportunities for agency, growth, and development that happen *at the individual level*. Although collective experiences are key for empowering processes in early adulthood, the model focuses on developmental conditions and holistic implications for *individual* empowerment.

The present empowerment theory model was constructed following the steps described by Peterson (2014). The author states that the first step in constructing a conceptual model of empowerment is clarifying its nature as a higher-order multidimensional construct. In the theoretical model, youth empowerment is an aggregate construct, implying that: (a) the direction of causality goes from the measures to the construct; (b) each dimension is capturing a different aspect of empowerment. Additionally, as the dimensions are latent constructs themselves (first-order constructs), it configures an aggregate model of empowerment—a multidimensional construct formed by its dimensions (Peterson, 2014).

The current model conceptualizes integrated empowerment in the transition to adulthood as a systemic, multilayered process. Based upon the person-context view, the model considers that youth empowerment does not constitute an inherent trait or a process solely external to the individual. Instead, the empowerment process emerges through the active, ongoing interaction between the individual and the relational environment. In an iterative process, the young person goes through experiences in context, and through self-reflection and feedback from others, he/she reiterates and further develops the interlinked dimensions.

**Figure 1:**
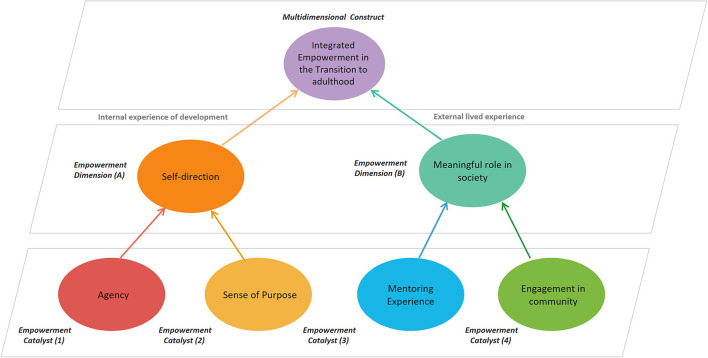
Model of the integrated empowerment theory: transition to adulthood.

Empowerment emerges as the interplay between two foundational dimensions. Representing internal processes of development, the key catalysts personal agency and sense of purpose interact to produce the first empowerment dimension: the constitution of self-direction. Since internal development needs a “stage”, a concrete space for enacting and consolidating developmental gains, a necessary counterpart would be external experiences of development. In these, the key catalysts mentoring experiences and engagement in community come together to form the second dimension of empowerment: developing a meaningful role in society. These two dimensions combine and emerge as the overarching construct of empowerment.,). Our theoretical model of developmental empowerment in the transition to adulthood is presented in Figure 1.

### 4.1. Internal experience of development: self-direction

The first dimension refers to key developmental processes that occur internally to the individual and exist in a process of mutual influence with external lived experiences. Early adults engage in role experimentation and identity exploration to fuel key developmental catalysts*: personal agency* and *sense of purpose*. Together, they shape empowerment by assigning meaning and connecting experiences, allowing youth to develop self-direction (see Figure 1).

Self-direction involves processes of increasing self-knowledge and ability to reflect and make conscious and active decisions. This internal process is important in meeting the challenges of adulthood and taking responsibility for one's life. Self-direction is closely linked to self-authorship, to the extent that it involves the capacity to make decisions in which one's internal voice coordinates external influence (as described by Baxter-Magolda and Taylor, 2015). Gradual construction of self and alignment with purposeful directions in life foster processes of self-determination and exercise of choice in experimentation.

Earlier models of youth empowerment recognize that empowering processes may generate increased positive choices and behavior (Chinman and Linney, 1998), confidence and actualization of potential (Cargo et al., 2003). However, these works tend to focus on adolescents, particularly on improving decision-making processes related to risky/unhealthy behavior in teenage. The present model focuses on developmental needs that generally occur slightly later in the life span: the need to build capacities for independent decisions related to the transition to adulthood.

The *self-direction* dimension in the model is similar to what Martínez et al. (2017) described as the intrinsic power or learning dimension of empowerment: developing personal capacities and means to increase “power over oneself”. It is also linked to three features of youth empowerment described by the authors^4^: personal growth and wellbeing (conditions for healthy development); educational (competences, self-efficacy, and critical thinking); and emancipative (ability, authority, and confidence to make decisions and affect change) (Martínez et al., 2017).

Combined, *personal agency* and *sense of purpose* contribute to the development of *self-direction* as an increasing capacity to make thoughtful, self-authored, and strategic life choices at this stage of life. The next section advances the conceptualization of these two catalysts to the internal experience of developmental empowerment.

#### 4.1.1. Catalyst 1: personal agency

The prior literature in the empowerment field includes a wide range of variables or conceptual terms that capture the internal, personal, or psychological indicators. Following is a list of those terms cited in the literature, accompanied by a critical appraisal of their appropriateness for a model of developmental empowerment in early adulthood.

(a) Self-esteem (Chinman and Linney, 1998; Cargo et al., 2003; Morton and Montgomery, 2013; Martínez et al., 2017). Self-esteem is a broad and generic concept reflecting one's global evaluation of self-worth based on beliefs and subjective emotions. Although increasing levels of self-esteem in youth may be associated with empowering processes, the concept seems ill-suited to be by itself an accurate description of internal development or a clear indicator of progress in these processes, given that the nature of empowerment is fundamentally context-driven, multi-faceted, and action-based.
(b) Self-efficacy/beliefs about competence (Rappaport, 1987; Zimmerman, 1995; Chinman and Linney, 1998; Cargo et al., 2003; Jennings et al., 2006; Cattaneo and Chapman, 2010; Wong et al., 2010; Morton and Montgomery, 2013; Krauss et al., 2014; Peterson, 2014; Martínez et al., 2017). Self-efficacy denotes one's beliefs in his/her ability to achieve goals and power to affect situations. Although narrower and more focused than the previous concept, self-efficacy by itself is not a sufficient indicator of internal development in empowering processes to the extent that it is limited to individual beliefs that may or may not promote a person's effective capacity to affect and master his/her environment.
(c) Self-confidence and mastery (Rappaport, 1987; Chinman and Linney, 1998; Cargo et al., 2003; Cattaneo and Chapman, 2010; Krauss et al., 2014; Peterson, 2014). Confidence and mastery are fundamental in the original concept of empowerment. Referring to a sense of self-assurance in personal ability, power, and judgment, self-confidence is intrinsically linked to experiences of mastering particular activities. Mastery refers to a process of learning skills and gaining control over personal matters. The concepts are close to self-efficacy, but with the advantage of being linked to concrete experiences of empowerment. Although those are essential aspects, they are not appropriate as sole indicators of internal empowerment. It would be necessary to consider their interaction with other indicators such as autonomy in decision making, self-reflection, and sense of control, for example.
(d) Knowledge, intellectual competence, and understanding of socio-political environment (Zimmerman, 1995; Chinman and Linney, 1998; Cargo et al., 2003; Cattaneo and Chapman, 2010; Wong et al., 2010; Krauss et al., 2014; Peterson, 2014; Martínez et al., 2017). Many authors recognize cognitive factors and knowledge about the environment as keys to increased empowerment as individuals develop resources to influence and control their destiny. These factors are especially salient in empowerment models aimed at socio-political and community change. Although the approach in the present study recognizes the importance of these factors (e.g., ability and willingness to seek information about occupations is fundamental to empowering processes in career exploration), they are not particularly salient in that the focus is on personal development in early adulthood.
(e) Awareness, self-reflection, and critical consciousness/thinking (Freire, 1970; Chinman and Linney, 1998; Cargo et al., 2003; Jennings et al., 2006; Martínez et al., 2017). Ability to understand oneself and others, recognize one's thinking processes and how they affect behavior and outcomes, and develop one's unique perspective while “reading” the exterior environment and reflecting about the world—these are key factors for developmental empowerment in this stage of life. Self-reflection is the capacity for introspection and learning about one's nature and actions. Critical consciousness refers to an in-depth understanding of the world. These concepts are central to allowing youths to integrate diverse experiences into their whole development—and therefore an essential component of personal agency. However, by themselves, they are not sufficient to comprehend the internal experience of empowerment. Instead, they would be appropriate if included in a composite measure.
(f) Autonomy in life decision-making/self-determination (Chinman and Linney, 1998; Cargo et al., 2003; Jennings et al., 2006; Cattaneo and Chapman, 2010; Krauss et al., 2014). Decision-making refers to the identification and choice of alternatives based on one's preferences and values. Autonomy refers to freedom to determine one's own actions and behavior. Self-determination also points to free choice of acting without external coercion. These are essential notions in the construct of personal agency, and therefore indispensable indicators on a model of personal empowerment, in combination with other factors.
(g) Sense of control and predictability in life/sociopolitical control (Zimmerman and Zahniser, 1991; Zimmerman, 1995; Chinman and Linney, 1998; Cattaneo and Chapman, 2010; Wong et al., 2010; Peterson, 2014). Sense of control refers to beliefs about one's control over the outcome of events, as opposed to external forces. Sociopolitical control refers to the degree to which the individual believes he/she is capable and effective in social and political systems. Intrinsic control is a key aspect in the notion of empowerment and should be considered in a measure of the internal experience of development. However, the agency construct is more effective in application of empowerment in the transition to adulthood because it adds to the sense of control a dynamic, forward-leaning quality that allows for the accomplishment of personal goals.

Our conceptualization, in turn, rather defines personal agency as the construct able to capture the internal, personal, or psychological indicators of empowerment in the transition to adulthood. The agency construct aggregates the main aforementioned concepts in a dynamic and self-determined construct.

Agency refers to the “ability to exert influence on one's life” [Macmillan's review of theory and research on agency (Macmillan, 2006)]. Empirical findings (Schwartz et al., 2005; Krauss et al., 2014) allowed to extend the definition of agency to include: perceived control of one's decisions and confidence to overcome obstacles; and beliefs about one's abilities to set goals and organize actions to achieve those. Increasing autonomy, self-knowledge and reflection are also fundamental components. Findings from research (Schwartz et al., 2005) demonstrated that early adults who address issues proactively are most likely to form a coherent sense of identity that will guide their life paths and facilitate the transition to adulthood. Agency is “positively related to exploration, flexible commitment, and deliberate choice making” (p. 222).

The empowerment model proposes a global measure of agency (Personal Agency) which integrates several concepts (named Agency in the Transition to Adulthood Scale), mapped in the nomological network presented on Table 1.

**Table 1:** Nomological network for the construct personal agency: agency in the transition to adulthood scale.

**Concepts and indicators**	**Items in the measurement scale**	**Sources**
Confidence and mastery	I am confident in my ability to set goals and work to achieve them	Krauss et al., 2014
Self-awareness and self-esteem	I have a strong sense of self and identity	Schwartz et al., 2005
Awareness, reflection, and critical consciousness	I reflect about the world and the sociopolitical context	Zimmerman, 1995
Sense of control and confidence	My experiences and actions are under my control	Polito et al., 2013
Autonomy in decision-making, self-determination	I am increasingly becoming self-directed and independent	Steinberg, 2007
Self-understanding and self-reflection	I increasingly understand myself	Steinberg, 2007

#### 4.1.2. Catalyst 2: sense of purpose

Youth is a formative period for cultivating a sense of purpose, which is recognized as a vital indicator of adolescent thriving, as research shows its proactive, defensive, and healing roles for youth (as stated by Damon et al.'s comprehensive review of theory and empirical research on development of purpose in youth, 2003). Malin et al. (2014) further demonstrated in a cross-sectional study that youth purpose changes over time. In middle school, youth desired to be empathic; in high school, their focus was on finding a role to engage their purpose; and in college, youth focused on developing pathways to support their purpose.

Considered a “central driving force of personal growth and social change” (Damon et al., 2003, p. 119), purpose is defined as a “stable and future-oriented intention to accomplish something that is meaningful to the self and to the world beyond-the-self” (p. 121), accompanied by an active engagement to realize it. Sense of purpose is also a fundamental factor in the process of engagement and participation, considered as a key component on the pathway of creating meaningful trajectories in life and successful transition to adulthood.

Sense of purpose is largely absent in earlier models of empowerment, with two exceptions. Chinman and Linney (1998) point to the importance of purpose when they elaborate on the centrality of meaningful activities at work for adolescents. However, the authors do not address life purpose in a broader sense, but refer to a narrower concept, limited to immediate realities in adolescence. Cattaneo and Chapman's (2010) model somehow encompasses a personal sense of purpose, as it frames empowerment as intrinsically linked to personally meaningful goals, through experiences that are energized solely in particular contexts, related to their meaning to specific people.

Our approach considers sense of purpose as a fundamental aspect in empowering internal experiences of development, as it naturally balances and complements personal agency. A sense of purpose assigns meaning to and connects the experiences toward integration and realization of the youth's potential.

The classic book by Frankl (1959) presented the personal search for meaning as a primary force and a key to achieving integrity and fulfillment in life. The search for meaning is considered an inward reflection (Malin et al., 2014). Especially in youth, finding meaning in life and developing an authentic sense of self are fundamental aspects for integral development (Rossiter, 2001). Value autonomy begins to emerge in adolescence, progressing toward a roughly coherent and consistent set of attitudes [Adelson's theory on adolescent political thinking (Adelson, 1971)]. Adolescence and early adulthood are key periods on the constitution of values that will roughly define the path individuals will follow through the rest of their lives.

Desire for “changing the world” is a hallmark of adolescence and early adulthood. Young people often present positive and promising interests and characteristics that can be supported and encouraged, such as: motivation to create innovative practices, aspiration for change and “desire to connect with or contribute to something larger than the self” (Malin et al., 2014, p. 188). As conceptualized by Damon et al. (2003), the sense of purpose does not supersede self-interest; the aspiration integrates self and beyond-the-self interest.

A healthy and meaningful transition to adulthood might include the exploration of purpose-driven career goals, combining individual trajectories with a beyond-the-self purpose and leading to a more comprehensive and coordinated way of life with community. The quest for career definition benefits from identifying a purposeful drive to contribute to society in a meaningful way.

Youth's emerging capacity for theoretical thinking is allied with the natural aspiration for change, resulting in an ability to create strong and bright visions of possibilities for the future. Envisioning better futures, a sense of optimism and motivation for service can be nurtured as an integral part of self-development and healthy outcomes in the transition to adulthood. However, keeping a purpose-driven focus can be challenging in contemporary society, which by principle values other orientations (e.g., personal success, financial rewards, and extreme individualization) and presents several competing priorities to early adults.

Our model proposes a nomological network and psychometric scale for the Sense of Purpose construct (Youth Purpose Scale), which can be consulted on Table 2.

**Table 2:** Nomological network for the construct sense of purpose: youth purpose scale.

**Concepts and indicators**	**Items in the measurement scale**	**Sources**
Search for meaning and purpose	My life has a clear sense of purpose	Schulenberg et al., 2011
	I have discovered a satisfying life purpose	Schwartz et al., 2005
Orientation to contribute to greater good	I want to make a positive difference to the community around me and to the world	Damon et al., 2003
	It is important to me to help solve problems beyond my own life	Malin et al., 2014

### 4.2. External lived experience of development: meaningful role in society

The second dimension, developing a *meaningful role in society*, addresses external lived experiences of development, fundamental to opportunities for experimentation and growth. Catalysts are *mentoring experiences* and *engagement in community*. They offer a “stage” for youth to enact the internally developed processes in real-world settings and in participation with other youth and adults in different roles and forms of interaction (see Figure 1).

Conceptualizations of empowerment usually include external experiences of engagement in context: people developing a sense of community and commitment (early theory on empowerment by Rappaport, 1987); people exercising extrinsic power in opportunities for action and gaining control over the environment (review of empowerment conceptualizations by Martínez et al., 2017); relationships as essential aspects for psychological empowerment (theory on relational components of psychological empowerment by Christens, 2012); youth participating in collective efforts, as agents of both individual and community change [critical model of youth empowerment by Jennings et al. (2006); typology of youth participation by Wong et al. (2010)].

The development of youth's consciousness and active exercise of its place and role in society is a fundamental dimension of empowerment in early adulthood. It is a goal oriented to the future and has the potential to define not only the individual course but to shape the course of the society as a whole. As mentioned, the core of youth self-construction is based on interpretation of oneself and of one's world. Reviews on theory and practice in adolescent development (Nakkula and Toshalis, 2006; Curtis, 2008) affirm that as adults have an enormous impact on those interpretations, the way they guide young people today will determine the world's future.

Personal interactions with young people are a training ground for the roles they will assume in society in the future. Chinman and Linney (1998) emphasize the importance of promoting adolescence “role training” for a meaningful role as adults, as they recognize rolelessness as a major developmental risk. Cargo et al. (2003) also position youth participation in community as preparation for future roles, socially perceived as an “embrionary stage” of participation.

Engagement in community is a way to provide youth with the context, the experiences and the tools to improve a meaningful development process toward adulthood. Participants in community processes are affected by their participation: there are changes in identity, ownership, and locus of control, due to the opportunity to be in a dialogical process and possibly affect social change.

Combined, *mentoring experiences* and *engagement in community* offer context, resources and opportunities for youths to constitute a *meaningful role in society* in their future trajectories.

#### 4.2.1. Catalyst 3: mentoring experience

Early conceptualizations of empowerment elaborated on the role of facilitators in empowering processes: understanding and creating conditions to permit change, and enabling others to become empowered (Rappaport, 1987). Since the origin, facilitation and collaboration were considered parts of developmental processes of empowerment [Kieffer's theoretical framework for citizen empowerment (Kieffer, 1984)]. Contemporary works also elaborate in the role of people who “motivate and guide others in processes of collaborative and shared leadership” (Christens' theory on relational components of empowerment, 2012, p. 118), advocating for the need to better understand the role of facilitators in empowerment processes (Cattaneo and Chapman's empowerment framework, 2010).

Mentoring and multigenerational partnerships are described by several authors, as empowering processes are conceptualized as transactional partnerships and developmental alliances between adults and youth [youth empowerment model by Cargo et al. (2003); review of theory and practice by Nakkula and Toshalis (2006); typology of youth participation by Wong et al. (2010); empirical evidence in Krauss et al. (2014)]. The role of mentoring and peer approval in youth empowerment is also highlighted by Chinman and Linney (1998) model, as it states that mentoring leads to meaningful roles and developmental outcomes.

Adults create contexts for young people to gradually take responsibility, offering a welcoming environment and support for efficacy and mattering through mentoring and providing feedback [youth empowerment model by Cargo et al. (2003); empirical findings by Krauss et al. (2014)]. The role of adults as guidance, supervision, and social support is considered by Wong et al. (2010) empowerment framework, as it describes a continuum of youth-adult involvement. Jennings et al. (2006) highlights the importance of welcoming, safe, and supportive environments, in which youth experience shared power with adults in a delicate balance. A review of research on developmental gains in youth empowerment programs (Morton and Montgomery, 2013) shows that in ideal empowering processes, adults act as facilitators allowing high autonomy of youth.

Empowering mentoring initiatives take into consideration youth's needs, interests, and abilities. Evidence from a prior study (Krauss et al., 2014) showed that intergenerational partnerships that truthfully consider youth voice and perspective contribute to increased agency, empowerment and community connections. Providing contexts and opportunities for youths to express their voice creates positive perceptions both of youth and of adults. A review of practical applications with youth (Curtis, 2008) affirms that adults that value youth should show/model how to do it: listen to young people, explore their strengths, interact with them and appreciate their input. The structure for participation might be configured to encourage young people to actively collaborate, which contributes to critical dialogue and promotes awareness.

However, youth mentoring experiences are not inherently beneficial, and a cautionary view should be put in place (McCluskey et al.'s literature review on mentoring, 2004). Findings of an analysis of the effects of youth mentoring programs (DuBois et al., 2002) demonstrate that outcomes for youth at-risk vary substantially, with potential for ill-conceived or ill-implemented programs to actually have an adverse effect. Mentoring programs' positive effects are significantly enhanced with well-planned design, implementation, and assessment, which should fully address contextual factors and the need to build strong relationships between mentors and mentees.

Guidance, support, and appreciation are key aspects in mentoring experiences. Mentors provide support by listening, advocating, sharing resources, providing structure, highlighting strengths, and promoting positive experiences (McCluskey et al., 2004). Mentoring is a key process and context for empowerment in early adulthood, as authors point that the quality of relationships plays a critical role in empowering processes (Christens' theory of relational components of empowerment, 2012), and supportive adult relationships are key for developmental settings (Krauss et al.'s empirical evidence, 2014). Guidance and support are even stronger when combined with a sense of equity and balanced power between adults and youth.

Inviting youth to work alongside adults to make decisions fosters an overall sense of connectedness, which in turn contributes to desirable developmental outcomes like competence, self-efficacy and sense of control—as stated in Wong et al.'s (2010) typology of youth participation and supported by evidence from a youth engagement program reported by Browne et al. (2011).

The empowerment model proposes the construct of Mentoring Experiences to be assessed through a measure integrating concepts mapped in the nomological network shown at Table 3 (Youth-Mentor Relationship Scale).

**Table 3:** Nomological network for the construct mentoring experiences: youth-mentor relationship scale.

**Concepts and indicators**	**Items in the measurement scale**	**Sources**
Guidance	My mentor helps me understand the steps toward success	Wu et al., 2014
Power sharing	I felt my mentor values my voice, we exchange ideas and work collaboratively	Wu et al., 2014
Support	I feel supported by my leader or mentor	Scaffolding concept in Vygotskian theory (Vygotsky, 1978)
Appreciation	My leader or mentor acknowledges my work and contributions	Scaffolding concept in Vygotskian theory (Vygotsky, 1978)

#### 4.2.2. Catalyst 4: engagement in community

Participation in community efforts is likely the most traditional dimension of empowerment since the concept's inception. Empowerment is described as an essentially participatory competence by Kieffer [citizen empowerment framework (Kieffer, 1984)], while Rappaport [community empowerment theory (Rappaport, 1987)] elaborates on how the conditions of participation impact empowerment of members. Even the study of psychological empowerment originally considered the centrality of community participation, as Zimmerman (1995, 2000) stated that people empower themselves through participation in activities and organizations, and that empowerment at an individual level includes collective experiences of decision-making and problem-solving in the environment.

More recent empowerment theories also highlight the importance of social context and culture (Cattaneo and Chapman, 2010), with participation in collective change as an important medium for mutual empowerment, creating connections, developing collaborative competences, and passing legacy (Jennings et al., 2006; Christens, 2012; Krauss et al., 2014; Martínez et al., 2017).

Evidence from research on youth empowerment programs (Browne et al., 2011; Morton and Montgomery, 2013) demonstrate that engaging youth in community issues has important implications leading to developmental outcomes such as independence, problem-solving, hands-on learning, leadership, increased sense of control and personal responsibility, and overall wellbeing. Active community participation is considered a way to empower youth for proactive and healthy behaviors (protective/preventive) (as stated in Cargo et al.'s empowerment framework, 2003). Findings from research (Krauss et al., 2014) show that increased participation leads to gains in agency and confidence. Studies of community-based projects with youth (Lakin and Mahoney, 2006; Chaskin, 2009; Krauss et al., 2014) demonstrate that empowering settings are those that promote a sense of community, encourage cooperation, collaborative decision making, and frame the participation of young people as active, autonomous, responsible social actors and agents of change in their own right.

Meaningful participation addresses youth's intrinsic motivation to participate. Opportunities to engage in community through activities that are relevant to their lives, authentic and youth-determined promote youths' engagement and ownership, then contributing to development. Engagement is increased when the work is related to their experiences, authentic, interesting, fun, and relevant to the real world as they perceive it. Research shows that creating environments for young people to define their own motivations and drivers for action is a promising strategy (Mouchrek, 2014). Early youth empowerment theoretical models (Chinman and Linney, 1998; Cargo et al., 2003) affirm that meaningful participation in activities and experience with roles and responsibilities leads to developing skills, bonds, and recognition. Youth's meaningful participation allows affirming identity and nurturing social relations with the community.

Youth engagement in the community should be configured as a full partnership, where the members “learn as one”. Change-oriented work and learning dissolves “us and them” mindset, creating knowledge toward shared goals (Harder et al., 2013). Theoretical frameworks of empowerment and participation (Chinman and Linney, 1998; Cattaneo and Chapman, 2010; Wong et al., 2010) position mentors and facilitators as co-learners in collaborative empowerment processes: relationships in which all members learn from and support one another and raise the level of collective consciousness.

In processes of mutual learning, ownership is equally distributed, and members articulate perceptions and improve capacities to envisage future change together. Shared decision making can build skills, mastery and competence (Wong et al., 2010). Studies of youth community participation (Eccles and Gootman, 2002) show that community programs aiming to develop co-learning processes with youth must map onto their growing maturity and expertise, increasing cognitive capacities, increasing concerns about identity and movement toward adulthood. Processes of shared construction and co-learning are also promising strategies to contribute to changing the perceptions about youth in a positive way.

Our empowerment model proposes a nomological network for the Community Experience construct (Youth-Community Experience Scale), reported on Table 4.

**Table 4:** Nomological network for the construct community experiences: youth-community experience scale.

**Concepts and indicators**	**Items in the measurement scale**	**Sources**
Sense of belonging and actualization	For me, there is a strong feeling of belonging to this community	Maslow, 1962
	I undertake activities in that community that were meaningful and valued	Maslow, 1962
Social capital and social identity	I feel like I collaborate in creating the success of the common good of the organization/community	Tajfel and Turner, 1986; Coleman, 1988; Putnam, 2000
	The organization/community effectively allows me to use my talents, skills, and potential	Tajfel and Turner, 1986; Coleman, 1988; Putnam, 2000

### 4.3. Empowerment process

Because development occurs in context, empowerment emerges through real-world experimentation and active engagement. This conceptual model illustrated in Figure 1 proposes that the way empowerment develops from engagement experiences depends upon four key catalysts: agency, purpose, mentor, and community. The catalysts of agency and purpose represent two aspects of the internal experience: internal processes and empowerment. Agency provides the fuel for engagement and is sustained by a self-perception of faith in handling tasks and unknown challenges that arise through engagement. Purpose gives direction to the fuel of agency. Purpose matures from the desire and commitment to pursue a future goal state, which in turn serves to organize one's intentions and actions. Through self-reflection and meaning making in relevant activities, individuals develop agency and purpose, twin psychological processes that characterize an internal context that encourages empowerment.

The internal processes of agency and purpose occur in situations and contexts within a person's environment. In this model, the key catalysts of these external contexts are mentoring and community. Both aspects of the external context intersect with the individual's development. Effective mentoring enables the discovery of purpose. Through engagement with mentors who accompany rather than direct, youth encounter their own yearnings by engaging in guided experiences. Similarly, when mentors acknowledge and value the contributions, youth sense of agency is supported, and self-direction is enhanced. Through community youth gradually develop self-direction, independence, and capacity to envisage future change. Together these catalysts create a dynamic process with concrete outcomes, resulting in a growing ability to take meaningful, self-authored, and concrete actions, and promote effective change in life situations.

The model of empowerment in the transition to adulthood is not limited to normative developmental processes. The model does not propose the development of the four catalysts (agency, purpose, mentoring, and community) at the same time, nor with the same intensity. The idea is that all four aspects are equally important for empowerment and that interventions to promote empowerment should seek to balance these aspects. Some populations will naturally have one or another more developed aspect—the fact that interventions are participatory ensures that participants learn together and balance each other's factors.

Individual trajectories present great variability and the four catalysts might be constituted in different ways, which include both expected developmental stages and non-normative processes, such as resilience and grit, for example. The model accounts for interaction between the empowerment process and these other important developmental processes.

Resilience is a dynamic process of positive adaptation with multiple outcomes within the context of significant adversity (Luthar et al., 2000). It is possible to consider that some individuals might present strengthened personal agency and/or a deep sense of purpose as a result of their adaptive functioning. In the opposite direction, empowering settings and experiences (like mentoring and participation in community) may have the potential to facilitate resilience. Ungar (2011) addresses evidence that resilience is not necessarily an individual trait but more a function of the social and physical ecology around a person—hence the centrality of a young person's culture and contexts. Resilience is shown to involve a developmental progression: new ways of functioning emerge with new life situations (Luthar et al., 2000). We hypothesize that resilience and empowerment may interplay in several potential points of contact, which will vary depending on the individual and on the context.

Another possible interaction occurs between empowerment and grit. Grit is defined as “perseverance and passion for long-term goals, which requires working tirelessly toward challenges, maintaining effort and interest over years despite failure and adversity” (Duckworth et al., 2007, p. 1087). Grit is considered an individual trait of high-achieving people. We hypothesize that individuals with high levels of grit will present high levels of personal agency and sense of purpose, although these attributes might be circumscribed to specific life domains. When considering interplaying with the empowerment process, it might be particularly important to integrate those individuals in activities addressing external and more communal experiences such mentoring and participation in community. In alignment with our own approach, Bonfiglio (2017) questions the recent emphasis on individual students' grit and resilience as sole indicators of college student success, advocating for less individualistic conceptions and for the need for interventions that foster students' connectedness, social bonding, and integration. The author states that, in order to navigate life as adults, students need to cultivate not only individual skills such as confidence and competence, but also collectivistic outcomes such as meaning, purpose, empathy and community.

### 4.4. Empowerment indicator measures: Personal agency, sense of purpose, mentoring experiences and engagement community

The theoretical model and its measurement scales were tested on an empirical study with undergraduate students at a public land-grant research university in the United States. Data were collected through an online survey questionnaire taking approximately 20 min. Recruitment emails were sent to students through approximately 70 faculty and staff members teaching classes and working in undergraduate programs in several different fields and diverse years. Approximately 1,000 students received the email recruitment and 255 responded (25% response rate).

Participants were 255 early adult college students from all eight colleges (mean age of 19.4, 56% women/43% men, 40% first-year/33% second-year/27% third-year/10% fourth or final-year, 36% first-generation students). The research was designed primarily as a quantitative study, collecting data through an online survey questionnaire investigating the empowerment constructs and measures.

The questionnaire consisted of 26 questions, divided into three sections. Section 1 investigated participation in two recent important communities, with questions about quality and influence of mentoring and community experience. Section 2 approached personal agency, sense of purpose, life goals, and career identity status. Section 3 gathered demographic information about the respondents.

Across the four scales, age showed no significant contrasts. For gender, the only significant effects for higher scores for women, compared to men, on personal agency (*t* = 2.11, *p* < 0.05) and quality of community (*t* = 2.54, *p* < 0.05).

All four subscales in the Integrated Empowerment in the Transition to Adulthood Measurement Scale presented high reliability for the data in the study (consult Table 5 for descriptive statistics and reliability coefficients). The measurement scale items are described on Table 6.

**Table 5:** Means, standard deviation, and internal consistency for measures in the study.

**Measurement scale**	**Mean**	**SD**	**Internal consistency**
Personal agency	4.31	0.64	0.87
Sense of purpose	4.16	0.71	0.79
Quality of mentoring experience	4.27	1.10	0.96
Quality of community experience	4.31	0.88	0.94

All measures have responses choices ranging from 1 (strongly disagree) to 5 (strongly agree).

**Table 6:** Integrated empowerment in the transition to adulthood measurement scale (18-item).

**Subscales**	**Items**	**Internal consistency**
Personal agency	I am confident in my ability to set goals and work to achieve them. I have a strong sense of self and identity. I reflect about the world and the sociopolitical context. My experiences and actions are under my control. I am increasingly becoming self-directed and independent. I increasingly understand myself.	0.87
Sense of purpose	My life has a clear sense of purpose. I have discovered a satisfying life purpose. I want to make a positive difference to the community around me and to the world. It is important to me to help solve problems beyond my own life.	0.79
Mentoring experience	My mentor helps me understand the steps toward success. I felt my mentor values my voice, we exchange ideas and work collaboratively. I feel supported by my leader or mentor. My leader or mentor acknowledges my work and contributions.	0.96
Community experience	For me, there is a strong feeling of belonging to this community. I undertake activities in that community that were meaningful and valued. I feel like I collaborate in creating the success of the common good of the organization/community. The organization/community effectively allows me to use my talents, skills, and potential	0.94

The current scales serve only as indicators of the empowerment dimensions of self-direction and meaningful role. Trustworthy assessment of self-direction would require in-depth interviews, and full assessment of meaningful roles would require intensive ethnographic research with diverse mentors and communities. Overall assessment of the complex concept of empowerment is sufficiently complex to require in-depth case study analysis with extensive interview and observational data across multiple situations in time.

### 4.5. Application of the model

For youth practitioners wanting to foster active real-world engagement, the model advocates four strategies: establishing community, building agency, coordinating mentors, and inspiring purpose. In effectively establishing community, professionals and volunteers foster a shared sense of belonging and provide meaningful opportunities for youth to effectively use their skills and potential. When communities encourage confidence building and self-reflection, the context can bolster youth agency. Mentors who support and acknowledge the contributions of youth value their voice and preview paths to success. Interactions and shared constructions foster a sense of purpose, a desire to contribute to the greater good. Through co-learning processes with adults and peers, youth identity is shaped, and empowerment is activated. Up to this point, the sequence of catalysts presentation followed an alignment with the underlying theoretical model. The practice adaptation of the model uses a logical sequence of implementation of the four catalysts: Community, Agency, Mentors, and Purpose, or CAMP.

The flexible framework of CAMP has utility across a wide range of age groups exemplified by after-school programs in middle school, to high school clubs, to college leadership. The model similarly welcomes a broad array of content applications such as leadership programs, service learning, civic engagement, or career readiness. These diverse age and content applications intersect with many professions including teacher, counselors, university staff, and faith leaders, youth workers. For professionals looking to build programing from the model, one limiting aspect is that the catalysts are not well-suited for short-term intervention. Agency and purpose emerge through periods of development; mentoring and community are contexts that become established over time. Programs that seek to make meaningful, sustained change in the lives of young adults match the aims of the model.

The model hypothesizes that interventions aimed at supporting developmental empowerment in early adulthood have strongest potential when combining all four catalysts: stimulating agency, fostering reflection on purpose, offering opportunities for shared dialogue and action with adults, and positioning youths as potential active members in their communities. Interventions should be designed having in mind the characteristics of specific contexts, population, and developmental domains. Different needs will require different emphasis in the catalysts—and while having participants presenting different experiences might pose challenges in terms of the intervention design and implementation, on the other side diversity has great potential as a developmental asset in process of mutual learning and collective construction.

## 5. Future research

The intersection between the empowerment dimensions (self-direction and meaningful role) offers ample opportunities for future research.

(a) What happens, for example, when individuals have internal capacities such as agency and purpose, but lack the meaningful roles to express their self-direction?
(b) How do variations in mentoring and community contexts influence young adults' self-direction during the transition into adulthood?
(c) What role do self-direction and meaningful roles have in enabling the innovative potential youth can bring to societies for progress and renewal?

Additional questions for future research extensions include:

(d) How do features of social location (gender, class, age, race, ethnicity) intersect with a sense of self-direction and the access to supportive mentors and communities?
(e) How are the components of empowerment related to indicators of successful adjustment and positive mental health in early adulthood?
(f) How might these concepts be applied to other age groups such as self-direction among children or meaningful roles for aging adults?

Future development of research and intervention in diverse settings is needed to evaluate the appropriateness, applicability, and precision of the theoretical model.

## 6. Concluding comments

Empowerment, the power to choose, is a developmental process that requires both an experience of one's own self-direction and one's connection to meaningful roles in the social world.

Despite its possible broader application, the study suggests applying the model to college students, because universities have great potential to be empowering settings supporting youth's self-orientation and future plans, through intentional design of environments for development. College settings may provide opportunities for experimentation with varied roles, positive mutual learning processes, meaningful achievement, and reflection. Potential application of the model for intervention in varied settings suggest ways to positively influence the processes of personal, social, and civic identity formation for early adults, in particular in scenarios including college students with diverse cultural backgrounds and developmental needs.

For early adults, empowerment has important powerful implications for their societal contributions. Across the fields of art, science, technology, and beyond, early adults contribute importantly to formulating and disseminating cultural innovation. Classic theories of social change emphasize the innovative potential of youth across areas that include: new learning and the progression of culture Mead (1970), scientific discoveries that challenge existing paradigms (Kuhn, 1962/2012), and the power and energy of social movements (Hoffer, 1951/2002). In the absence of adult mentors and established communities, youth themselves construct meaningful roles with other peers, creating community and mentoring each other. The engagement in meaningful roles by early adult activists is evident in issues of climate change (Eide and Kunelius, 2021) and in democracy movements (Carey et al., 2021). Creating contexts where youth can play meaningful roles in their “emerging” social world holds positive potential for society.

## Data availability statement

The original contributions presented in the study are included in the article/Supplementary material, further inquiries can be directed to the corresponding author.

## Author contributions

NM conceived the initial idea and developed the theoretical model under the guidance of MB. MB supervised the research project, provided critical feedback and helped shape the research, and analysis and manuscript. NM wrote the manuscript in consultation with MB. All authors contributed to the article and approved the submitted version.

## Footnotes

^1^Some of Erikson's elements of identity resolution (1968).

^2^The present work is primarily oriented toward the third, fifth, and sixth vectors.

^3^Initially focused on prevention and risk, the Youth Development field is currently oriented toward the promotion of healthy development, capacity building, and empowerment (Jennings et al., 2006).

^4^The full list of empowerment features described by Martínez et al. (2017) is: “(a) personal growth and well-being; (b) relational; (c) educational; (d) political; (e) transformative; (f) emancipative” (p. 405).
