# Supplementary material for: The theory of integrated empowerment in the transition to adulthood: concepts and measures
Source: Front Sociol. 2023 Apr 17;8:893898. doi: 10.3389/fsoc.2023.893898 (PMC10149756; doi:10.3389/fsoc.2023.893898)
Supplement: Supplementary file 1 [file Table_1.pdf]

Appendix A

Table 1

Analysis of Prior Conceptualizations of Empowerment

**Theory/ Conceptualization**	**Author(s)**	**Main elements**	**Critical Analysis**
Psychological Empowerment	Zimmerman (1995, 2000)	Focus of empowerment is on capacity-building and participation of individuals in decisions that affect their lives. Interrelated components: (a) intrapersonal/emotional; (b) interactional/cognitive; (c) behavioral.<br>Direction of empowerment goes from the individuals to the world around them.	Addresses relevant components of empowerment at the individual level, but it does not offer a clear explanation of how the empowerment process happens.<br>Model does not help to explain how a progressive empowerment process would map into a developmental transition and help shaping internal experiences of development.<br>Model does not differentiate among life stages; hence it does not capture unique characteristics and challenges of emerging adulthood.
Expanded Psychological Empowerment	Christens (2012)	Expanded Zimmerman's model by adding a relational component (referring to mutual support, collaboration, collective exercise of transformative power to achieve change).<br>The relational component included collaborative competences, facilitating empowerment of others, and engagement in participatory behaviors.	Promising addition: the empowerment process is more clearly delineated: key role of relationships in empowering processes, focus on collaboration and mutual growth. Influence of participation in groups over personal processes is more evident; emphasis on the role of mentors and community.<br>Model remains limited in providing a deeper understanding of the empowerment process for each individual, considering both personal development and real-life experiences.
Adolescent Empowerment Cycle	Chinman & Linney (1998)	Holistic definition for youth empowerment; focus on developing meaningful roles for youth.<br>Opportunities for meaningful participation are central for experimentation and identity construction. Major developmental risk to be avoided is rolelessness.<br>Key aspects: dynamic partnerships, recognition from adults and approval from peers, time for self-reflection and personal understanding of experiences.	Foundational work in youth empowerment. Model applies fundamental concepts of adolescent development; strong focus on developmental gains from an empowering process planned and implemented by adults in youth programs.<br>Explain how adolescent participation in activities would lead to developmental outcomes (Social Bonding Development theory).<br>Because the transition to adulthood offers added challenges (changes in life contexts, relationships, capacity building for adult roles), we needed to further develop their model to capture emerging adulthood's nuances and more clearly explain the developmental and dynamic nature of the empowerment process between individual and environment.

**Theory/ Conceptualization**	**Author(s)**	**Main elements**	**Critical Analysis**
Youth Empowerment Framework	Cargo et al (2003)	Empowerment is defined as a transactional partnership between adults and youth. Active community participation is an essential factor.<br>Adults develop social contexts for youth to gradually take responsibilities – youth gradually engage, actualize potential, and cultivate constructive change.<br>Illuminates the nature of mentoring relationships and the developmental outcomes from intentional processes to promote empowerment among youth.	Foundational work, significant contribution to understanding processes of accompaniment and transfer of responsibility between adults and adolescents. However, the interventions as described are limited to community engagement programs for adolescents implemented by adults.<br>Foundation for a theoretical model of empowerment in the transition to adulthood, which should advance the description of additional challenges and opportunities for development at this stage of life.<br>Extended contexts of life and activity, coupled with the variety of mentors and growing autonomy in making meaning and navigating life in emerging adulthood call for advancing the model.
Critical Social Theory of Youth Empowerment	Jennings et al (2006)	Empowerment is a multi-level construct, a series of experiences in which youth, adults, organizations, and communities engage in collective action for social change.<br>Key dimensions: welcoming and safe environment; meaningful participation and engagement; equitable power-sharing between youth and adults; engagement in critical reflection on interpersonal and sociopolitical processes; participation in sociopolitical processes to affect change; integrated individual- and community-level empowerment.	Comprehensive model which advances and organizes essential elements of empowerment in previous theories, representing. Focus on the potential of young people to participate and influence social change.<br>Dimensions are well described. However, the framework is not explicit about the dynamic interaction among elements to produce empowerment at the individual level.<br>Model illuminates political dimensions of the process but does not fully capture the developmental dimensions. Empowerment of youth can exist without being necessarily directed to social change as the outcome.
Empowerment Model	Cattaneo &Chapman (2010)	Model incorporates both individual and social aspects and describes empowerment as an iterative process.<br>Key components: personally meaningful and power-oriented goals, self-efficacy, knowledge, competence, action, and impact.<br>Describe how individuals move through the process focusing on specific goals while reflection derives from experience.	Although not positioned as a model of youth empowerment exclusively, it supports understanding dynamic processes of empowerment, as they highlight the role of intrinsic motivation, reflection, and interaction with others.<br>Contributed to the description of the empowerment process, revealing its iterative and gradually-developing nature.<br>However, the focus on self-efficacy and specific goals does not align with the purposes of the present study, which aims to describe how the empowerment process essentially unfolds in different spheres of development in emerging adulthood.
